# Light therapy as a treatment of cancer-related fatigue in (non-)Hodgkin lymphoma survivors (SPARKLE trial): study protocol of a multicenter randomized controlled trial

**DOI:** 10.1186/s12885-018-4746-2

**Published:** 2018-09-10

**Authors:** Daniëlle E. J. Starreveld, Laurien A. Daniels, Heiddis B. Valdimarsdottir, William H. Redd, Jessie L. de Geus, Sonia Ancoli-Israel, Susan Lutgendorf, Catharina M. Korse, Jacobien M. Kieffer, Flora E. van Leeuwen, Eveline M. A. Bleiker

**Affiliations:** 1 Division of Psychosocial Research and Epidemiology, The Netherlands Cancer Institute, Plesmanlaan 121, 1066 CX Amsterdam, The Netherlands; 2 Department of Radiotherapy, Leiden University Medical Center, Albinusdreef 2, 2333 ZA Leiden, the Netherlands; 3 Department of Oncological Sciences, Mount Sinai School of Medicine, E 101st Street, New York, NY 10029 USA; 4 Department of Psychiatry, University of California, San Diego 9500 Gilman Dr #0733, La Jolla, CA 92093-0737 USA; 5 Department of Psychology, University of Iowa, E228 Seashore Hall, Iowa City, Iowa, 52241 USA; 6 Department of Laboratory Medicine, The Netherlands Cancer Institute, Plesmanlaan 121, 1066 CX Amsterdam, The Netherlands; 7 Department of Clinical Genetics, Leiden University Medical Center, Albinusdreef 2, 2333 ZA Leiden, the Netherlands

**Keywords:** Cancer related fatigue, Light therapy, Sleep quality, Randomized controlled trial, Hematology, Circadian rhythms

## Abstract

**Background:**

Cancer related fatigue (CRF) is one of the most prevalent and distressing long-term complaints reported by (non-) Hodgkin survivors. To date there has been no standard treatment for CRF in this population. A novel and promising approach to treat CRF is exposure to bright white light therapy. Yet, large scale randomized controlled trials testing its efficacy in these patients and research on potential mechanisms is lacking. The objective of the current study is to investigate the efficacy of light therapy as a treatment for CRF and to explore potential mechanisms.

**Methods/design:**

In a multicenter, randomized controlled trial we are evaluating the efficacy of two intensities of light therapy in reducing CRF complaints and restrictions caused by CRF in survivors of Hodgkin lymphoma or diffuse large B-cell lymphoma. Secondary outcomes include sleep quality, depression, anxiety, quality of life, cognitive complaints, cancer worries, fatigue catastrophizing, self-efficacy to handle fatigue, biological circadian rhythms of melatonin, cortisol and activity, and biomarkers of inflammation. We will recruit 128 survivors, with fatigue complaints, from academic and general hospitals. Survivors are randomized to either an intervention (exposure to bright white light) or a comparison group (exposure to dim white light). The longitudinal design includes four measurement points at baseline (T0), post-intervention at 3.5 weeks (T1), 3 months post-intervention (T2) and 9 months post-intervention (T3). Each measurement point includes self-reported questionnaires and actigraphy (10 days). T0 and T1 measurements also include collection of blood and saliva samples.

**Discussion:**

Light therapy has the potential to be an effective treatment for CRF in cancer survivors. This study will provide insights on its efficacy and potential mechanisms. If proven to be effective, light therapy will provide an easy to deliver, low-cost and low-burden intervention, introducing a new era in the treatment of CRF.

**Trial registration:**

The study is registered at ClinicalTrials.gov on August 8th 2017(NCT03242902).

## Background

After the introduction of modern radiotherapy and combination chemotherapy, Hodgkin lymphoma (HL) has become the prototype of a curable malignancy with cure rates of 80 to 90% [1]. Also, for selected patients with aggressive non-Hodgkin lymphoma, survival has improved significantly, i.e. the 5-year overall survival of patients with diffuse large B-cell lymphoma (DLBCL) varies from 40 to 85% [2]. Unfortunately, treatment of lymphoma is associated with various late adverse effects, including cancer related fatigue (CRF) [3].

CRF is defined as “a distressing, persistent, subjective sense of physical, emotional, and/or cognitive tiredness or exhaustion related to cancer and/or cancer treatment that is not proportional to recent activity and interferes with usual functioning” [4, 5]. Patients feel tired even after resting, have reduced capacity to carry out normal activities, experience slow physical recovery from tasks, and report diminished concentration [6]. CRF is one of the most frequently reported long-term symptoms in (non-) Hodgkin survivors with prevalence ratings between 25 to 60% compared to 10 to 25% in the general population [7, 8]. CRF significantly affects patients’ quality of life [5] and seems to be influenced by symptoms of depression, anxiety, and the presence of comorbid conditions [8].

Currently, there is no standard treatment for CRF. A range of non-pharmacological interventions to treat CRF have been investigated, including physical activity (PA), psycho-education, cognitive-behavior therapy (CBT), CBT with hypnosis (CBTH), mindfulness-based approaches, and a number of complementary and alternative medicine interventions (e.g., acupuncture/acupressure, yoga, music therapy) [5]. Some of these interventions, including PA [9, 10], CBT [11], and CBTH [12], have been associated with large effect sizes. In the case of CBT, these effects remain stable for at least 2 years [13]. These findings are promising but not without limitations. For example, motivation is essential to complete these interventions while fatigue can reduce the motivation for PA [14]. Also, CBT is labor intensive since it requires professional guidance for several weeks.

A new development in the treatment of CRF is the use of light therapy. During this therapy, patients are asked to expose themselves to bright white light (BWL) for 30 min within the first half hour after awakening. Systematic exposure to BWL was originally developed to treat seasonal affective disorder [15] and is currently the treatment of choice for this disorder [16–18] although a recent review provided less conclusive results [19]. Additionally, light therapy has been found to help restore circadian rhythm disturbances and sleep disorders [20, 21].

Several studies have investigated the efficacy of light therapy specifically for CRF. One study randomized breast cancer patients undergoing chemotherapy to either a BWL (*n* = 23) or a dim red light (DRL; *n* = 16) condition [22]. Results showed that the usual increase in CRF from baseline to the end of the fourth chemotherapy cycle was seen in women exposed to DRL, while such an increase was not seen in the group exposed to BWL. In addition, circadian rhythms became more synchronized and quality of life was better in the women exposed to BWL compared to women exposed to DRL. Another study used the same design to test the efficacy of light therapy for CRF in cancer survivors [23]. Results showed that fatigue decreased to normal levels in survivors exposed to BWL (*n* = 18) while survivors exposed to DRL (n = 18) stayed at clinically significant levels of fatigue. These results also showed a significant decrease in depressive symptoms and better sleep quality in survivors exposed to BWL compared to DRL. More recently, results were published from a larger RCT that included 81 cancer survivors [24]. Survivors exposed to BWL showed greater reductions in fatigue and improvements in mood, depressive symptoms and quality of life compared to survivors exposed to DRL. In summary, these findings support the use of light therapy as a treatment for CRF.

However, the mechanisms that explain the effect of light therapy on CRF have largely remained unexplored. Light is one of the strongest synchronizers of the circadian rhythm system [25]. When it enters the eye, light affects processes in the suprachiasmatic nucleus (SCN), a structure better known as the human master pacemaker of circadian rhythms [26]. Based on this knowledge, several hypotheses about potential mechanisms could be formulated.

The first hypothesis is that light therapy normalizes the sleep-wake cycle. Previous studies showed that sleep-wake cycles, measured with questionnaires as well as objective measurements with actigraphy, were disrupted in patients with cancer after chemotherapy and that this disruption was related to increased CRF [22, 27]. Furthermore, it was shown that light therapy during chemotherapy resulted in sleep-wake cycles that returned to baseline levels after chemotherapy while patients in the comparison condition showed disrupted sleep-wake cycles after four cycles of chemotherapy [27]. Moreover, secondary analysis on objective sleep data collected with actigraphy in cancer survivors with CRF suggested that exposure to bright white light improved the sleep efficiency to normal ranges while this improvement was not seen in the group exposed to dim red light [28].

The second hypothesis is that the mechanism may be related to changes in circadian rhythms. The superchiasmatic nucleus (SCN) is responsible for the production of melatonin, a hormone that is secreted in darkness, which acts as a time-cue for sleep. Melatonin shows a circadian rhythm with rising levels during the evening that reaches the peak during the night followed by a decrease that reaches its lowest point (nadir) in the morning. The SCN also plays a role in the production of cortisol, a glucocorticoid hormone that shows a sharp increase in the first 30 min after awakening, followed by a gradual decline over the day that reaches its nadir during the night [29]. Impairments of this rhythmicity, such as the flattened morning-rise and a lower ratio between morning and nocturnal levels of cortisol, have consistently been associated with deteriorations in mood in both healthy and clinical populations and increased CRF in clinical populations [30–32]. Light therapy was proven to be effective in entrainment of the circadian rhythms of melatonin and cortisol [33]. Moreover, improvements in CRF over time were associated with normalization of the circadian cortisol rhythm [34], suggesting that a potential mechanism of light therapy on CRF is via the normalization of the circadian rhythms of these hormones.

A third potential mechanism is the normalizing effect of light therapy on the HPA axis, which may affect inflammatory cytokine activity. There is a wealth of research, both in animals as well as in clinical and healthy human populations, showing strong interconnections between fatigue and inflammation. Consistent associations have been shown between CRF and plasma levels of inflammatory markers such as interleukin-6 and C-reactive protein [35, 36]. There is also a well characterized feedback loop between the HPA axis and inflammation, whereby the HPA axis can down regulate inflammation and is itself up regulated by inflammatory signaling [37]. BWL has been found to normalize HPA axis function [38] raising the possibility that BWL may affect inflammatory cytokine activity either directly or indirectly, e.g., via its normalizing effects on the HPA axis.

The main aim of this double-blind, randomized controlled trial, called ‘improving Sleep quality, Psychosocial functioning and cAncer Related fatigue with Light thErapy (SPARKLE)’, is to determine the effect of exposure to BWL compared to exposure to dim white light (DWL), on CRF in ≥2 years survivors of HL and DLBCL. Additionally, this trial will explore potential mechanisms of light therapy on CRF by investigating the influence of light therapy on factors associated with CRF. More specific, the secondary objectives are:

1. to examine the effect of exposure to BWL compared to DWL on sleep quality and psychological variables (depression, anxiety, cognitive complaints, and quality of life).
2. to investigate whether exposure to BWL, compared to DWL, affects circadian rhythms of cortisol and melatonin, activity, vitamin D concentrations and levels of biomarkers for inflammation markers.
3. to explore whether the effects of exposure to BWL on CRF can be predicted by the effect of BWL on sleep quality, psychological variables, biological and activity circadian rhythms, and inflammation markers.

## Methods

**Fig. 1:**
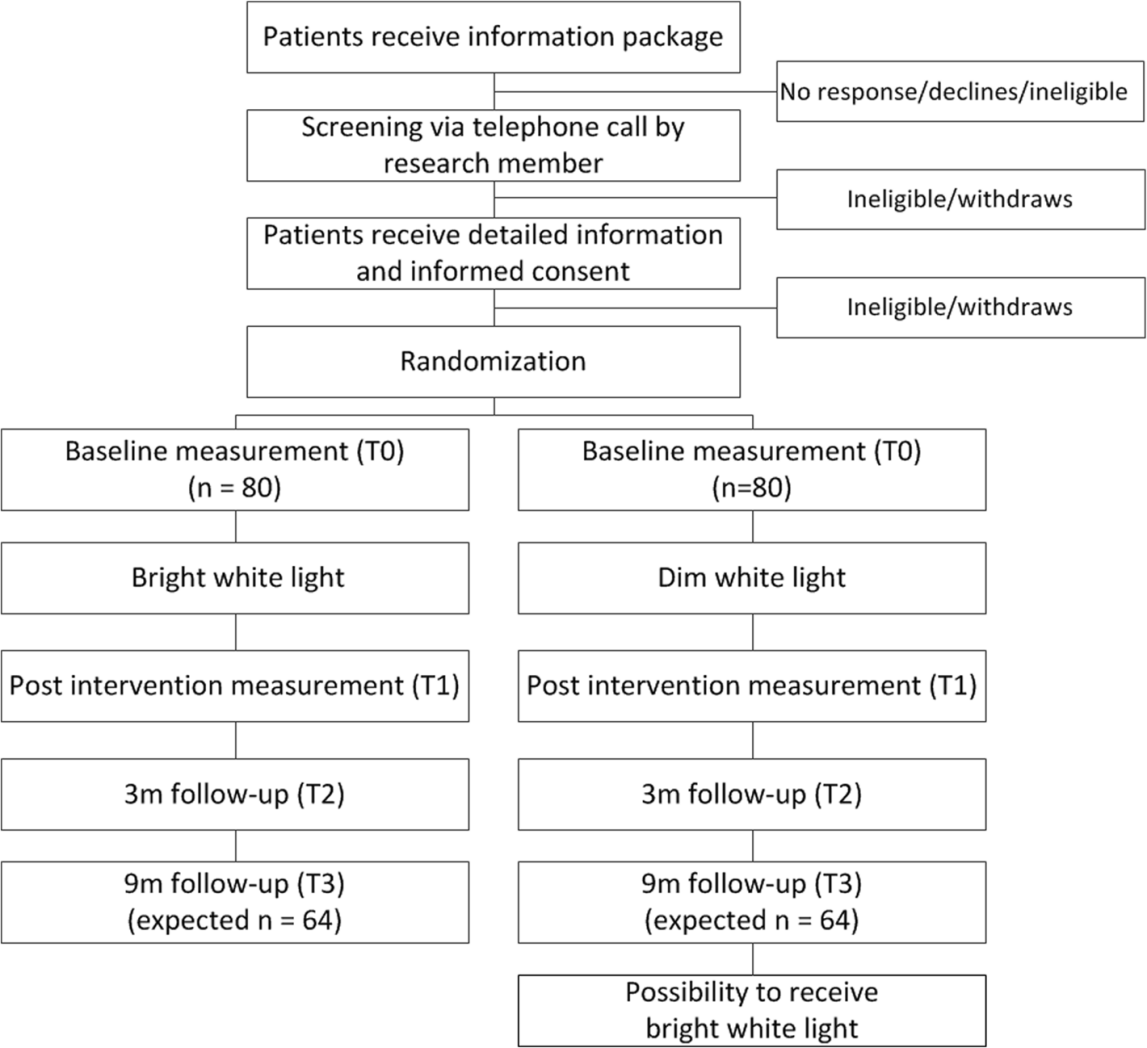
Overview of the trial design

This trial will use a double blind randomized controlled trial design with one intervention group exposed to bright white light and one comparison group exposed to dim white light. The design of the trial and the anticipated flow is shown in Fig. 1. This trial (under number NL61017.031.17) has been approved by The Institutional Review Board of The Netherlands Cancer Institute as well as by the review boards of the participating hospitals (see recruitment and randomization). Patient recruitment and data collection started in August 2017.

### Participants

The intended study sample will comprise 128 survivors of Hodgkin lymphoma (HL) or diffuse large B-cell lymphoma (DLBCL). Inclusion criteria are: (1) a survivorship of ≥2 years; (2) presence of moderate to severe fatigue symptoms since diagnosis of or treatment for HL or DLBCL. The presence of fatigue will be defined by fulfilling at least one of the following criteria: (a) a moderate to severe fatigue score on the general fatigue subscale of the multidimensional fatigue index; (b) a score of ≥17 on the Work and Social Adjustment Scale indicating clinical levels of impairments in daily functioning caused by fatigue [39].

Exclusion criteria are: (1) presence of somatic cause for fatigue (defined as (a) New York Heart Association class 3/4 (heart failure), (b) having a COPD gold class 3/4 (lung failure), or (c) having other organ failure that has led to marked limitation of physical activity). Patient can be included if, despite having used stable medication for ≥6 months for the somatic cause, fatigue complaints remain; (2) pregnancy (until 3 months postnatal) or lactating; (3) having had extensive surgery in the past 3 months; (4) having a current diagnosis of psychiatric disorder that can hamper participation; (5) having had a diagnosis of and/or treatment for secondary malignancy in the past 12 months; (6) presence of photophobia or other eye diseases that show symptoms of photophobia; (7) current or previous use of light therapy (≥ 1 week); (8) current employment in shift work.

### Recruitment

Participants for this study will be recruited via collaborating BETER-clinics. The BETER consortium (Better care after Hodgkin lymphoma: Evaluation of long-Term Treatment Effects and screening) is organising a nationwide infrastructure for survivorship care for lymphoma survivors, to prevent morbidity and mortality from late treatment effects [40]. This consortium identifies and traces 5-year survivors of HL and DLBCL treated in 23 Dutch academic as well as general hospitals. So far, eight BETER-clinics agreed to collaborate with the SPARKLE study: Antoni van Leeuwenhoek, LUMC, Radboudumc, VUmc, UMCU, ErasmusMC, Albert Schweitzer hospital, HagaZiekenhuis, Admiraal de Ruyter hospital.

Survivors (≥ 2 years) of HL or DLBCL who visit their treating physician for follow-up care are screened for CRF symptoms. When CRF symptoms are present and the patient meets the inclusion criteria, the physician will hand out a pamphlet, a response card and a screening questionnaire to the patient. A second strategy to recruit patients is via an evaluation of the BETER screening questionnaire that patients complete for their first BETER-clinic visit. This questionnaire includes a visual analogue scale (VAS) scale from 0 (no fatigue) to 10 (worst imaginable fatigue). If the fatigue score is 4 or higher, patients will be sent the information package.

Patients are asked to return the response card to express their interest in participation. In case of no interest, patients are asked to specify their reason(s) on the response card. If patients are interested, they are asked to complete the screening questionnaire and return this to the SPARKLE research team. Non-responders will receive a reminder 3 weeks after receiving the information package.

Patients who return the screening questionnaire receive a call from the SPARKLE research team. The aim of this phone call is to provide more information about the study and to screen on inclusion and exclusion criteria. Interested and eligible patients will receive a more detailed patient information letter and an informed consent form. Patients are requested to return a signed informed consent or a no-interest-response-card within 2 weeks. Non-responders will be called to assess willingness for participation 3 weeks after sending the patient information letter.

### Randomization

Equally distributed across all four seasons, participants are randomized to either an intervention group (*n* = 64) or a comparison condition (*n* = 64) using the minimization technique at a 1:1 ratio. Randomization is stratified for diagnosis (HL; DLBCL), time since diagnosis (< 5 years; 5–10 years; 11–20 years; > 20 years) and gender (male; female). Randomization is outsourced to an independent party, using the randomization programme ALEA. The output determines which lamp (with BWL or with DWL) is offered to each participant. This lamp will be part of the content of a bag offered to the research assistant who visits the participants. In this way, both the research team and the participants are blinded to the allocated condition. The randomization code will only be broken if a patient reports severe adverse side effects as a result of the light intervention.

### Description of interventions

Instructions for light therapy are equal in both conditions. All participants self-administer light therapy at home for 30 min each morning during a period of 3,5 weeks. Participants start with the light therapy within 30 min after waking up and position the light box at a distance of 45 cm and an angle of 45° from their face. During the light therapy participants can engage in other activities such as reading or having breakfast. They are informed not to stare into the light but to keep their eyes open to ensure that light falls on the retina. No instructions for sleep pattern adjustments are provided in the current trial.

Light therapy in both conditions will be administered via a Litebook© Edge (Litebook, Ltc. Medicine Hat, Canada). The Litebook© Edge is a small (15 × 12 × 1 cm), lightweight box designed to be placed on a table. The Litebook© Edge contains 60 premium white light emitting diode (LED) lights which mimic the visible spectrum of sunlight for minimum glare and maximum eye comfort. For purposes of safety, the Litebook© Edge emits no ultraviolet light. The Litebook© Edge devices used in this study were modified to include an integrated meter that allows for adherence monitoring by recording time and duration of on-time on each day.

#### Intervention group

The intervention group will be exposed to BWL with an intensity of 10.000 lx at a distance of 45 cm. The spectrum of the light in this condition will be enriched around 480 nm wavelengths. Light with this colour has previously been shown to be the effective factor in light therapy as it is associated with melatonin suppression [26].

#### Comparison group

Participants in the comparison condition will be exposed to dim white light, with an intensity of 10–20 lx at a distance of 45 cm. This light was successfully used as a comparison condition for BWL therapy in Alzheimer’s disease. Similar results are expected in cancer survivors [personal communication with Dr. M.G. Figueiro, November 14, 2016].

### Study procedure

**Fig. 2:**
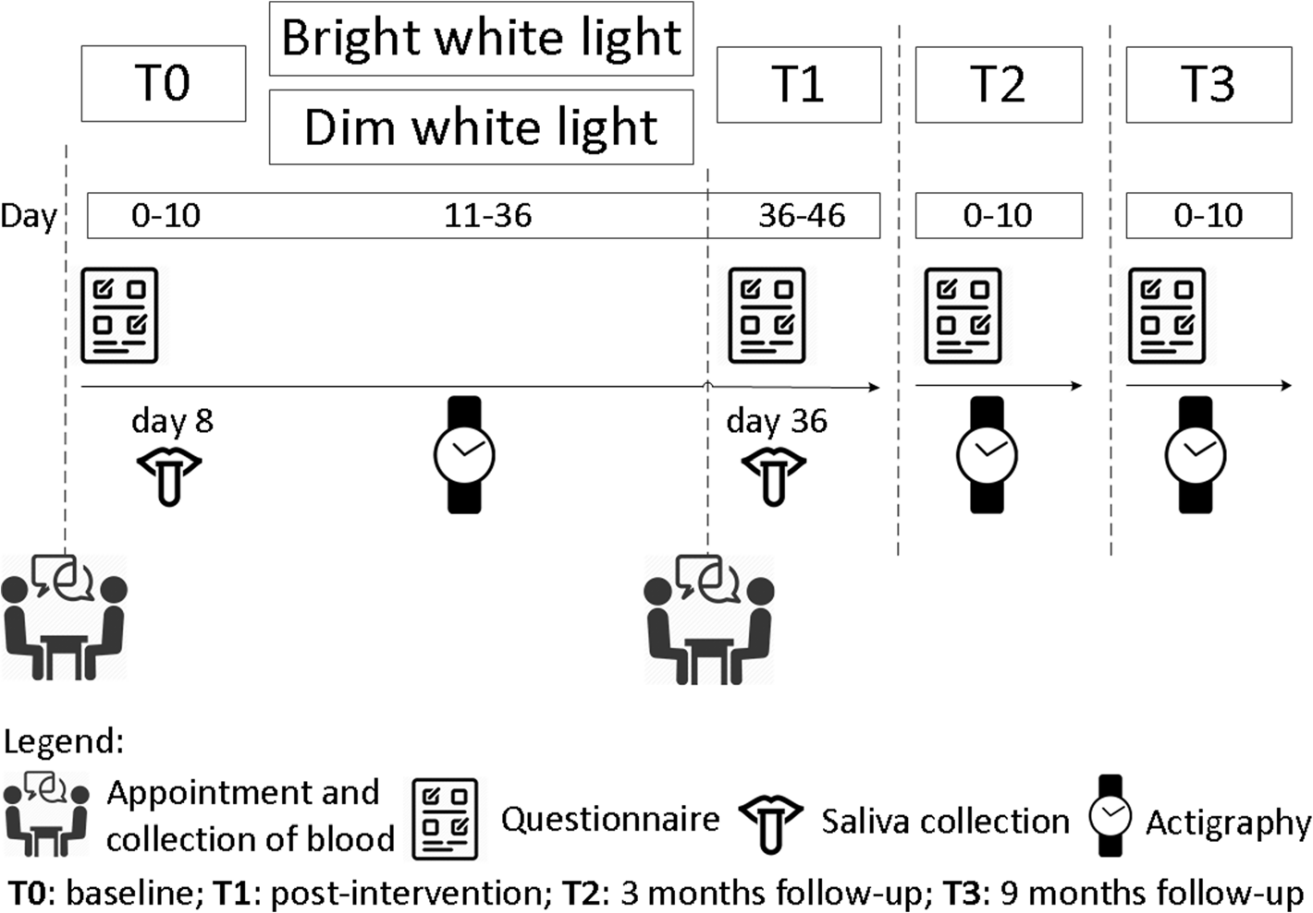
Overview of study procedure

All participants complete a battery of self-report questionnaires and wear a wrist actigraph at four different measurement points (T0: baseline; T1: directly after 3,5 weeks of light therapy; T2: 3 months after light therapy; T3: 9 months after light therapy). The first (T0) and second (T1) measurement points include a visit to the hospital to provide participants with materials and instructions, to perform cognitive tests, and to collect blood (during the visit) and saliva (on day 8 and 36) samples. Figure 2 shows a schematic diagram of a participant’s timeline.

The research assistant or study coordinator calls the participant after 5 days of light therapy asking for the occurrence of any side effects (headache, nausea, agitated feeling and irritated eyes). In normal cases, these side effects vanish in a few days. Light therapy is terminated when these side effects are still present after 5 days of light therapy. These participants are asked to complete all follow-up assessments.

After 3,5 weeks of light therapy, participants are asked not to use light therapy during the follow-up measurements. No instructions are provided for the use of concomitant care and other interventions.

### Study measures

#### Sociodemographic and clinical data

Information regarding the patients’ age, education, marital status, living situation, work status and medication use will be obtained via a questionnaire. Clinical information, including date of diagnosis, tumor characteristics, and treatment history will be abstracted from the BETER-database. This clinical information will be abstracted from the patients’ medical record when participants are not included in the BETER-consortium. Current season will be derived from the start date of light therapy.

#### Outcome measures

The Multidimensional Fatigue Inventory (MFI) [41], a VAS-scale for fatigue [42] and the Work and Social Adjustment Scale (WSAS) [43] are the primary outcome measure of this study. Secondary outcome measures include: Pittsburg Sleep Quality Index (PSQI) [44], wrist actigraphy [45], Center for Epidemiological Studies – Depression scale (CES-D) [46], State-Trait Anxiety Inventory-6 items (STAI-6) [47], Medical outcome studies short form (SF-36) [48], Medical Outcomes Studies Cognitive function scale (MOS-CF6) [49], MD Anderson Symptom Inventory (MDASI) [50], Psychomotor Vigilance Task (PVT) [51], 15 words task [52], digit span task [53], cancer worry scale (CWS) [54], fatigue catastrophizing scale (FCS) [55], Self-efficacy scale 28 (SES-28) [56], salivary cortisol and melatonin, and inflammatory biomarkers. Detailed descriptions of these outcome measures are provided in Table 1.

**Table 1:** Study outcome measures and corresponding questionnaires

Variable	Questionnaire	Number/type of items	Time frame	Score range	Psychometric details
Primary outcomes					
Cancer related fatigue	MFI [41]	20 four-point Likert scale	Past few days	Subscale scores: 4–20; higher scores indicate more fatigue	Subscales: general fatigue, mental fatigue, physical fatigue, reduced motivation, reduced activity. Cronbach’s alpha: 0.84
	VAS-scale [42]	1 eleven-point Likert scale	This moment	0–10; higher scores indicate more fatigue	
Restrictions caused by fatigue	WSAS [43, 70]	5 nine-point Likert scale	Influence of fatigue on daily life	0–40; higher scores indicate higher levels of disability	Cronbach’s alpha: > 0 .79
Secondary outcomes					
Sleep quality	PSQI [44]	19 four-point Likert scale and open-ended questions	Past month	Total score: 0–21 Subscale scores: 0–3; higher scores indicate more acute sleep disturbances	Subscales: subjective sleep quality, sleep latency, sleep duration, habitual sleep efficiency, sleep disturbances, use of sleeping medication, daytime dysfunction. Cronbach’s alpha: 0.83
Depression	CES-D [46, 71]	20 four-point Likert scale	Past week	0–60; higher scores indicate greater depressive symptoms	Cronbach’s alpha: 0.85–0.90
Anxiety	STAI-6 [47]	6 four-point Likert scale	This moment	20–80; higher scores indicate increased anxiety	Cronbach’s alpha: 0.83
Quality of life	SF-36 [48, 72]	36 Dichotomous three to six-point Likert scale	Past 4 weeks	Subscale scores: 0–100; higher scores indicates higher levels of functioning/well-being	Subscales: physical functioning, role limitations due to physical health problems, bodily pain, social functioning, general mental health, role limitations due to emotional problems, vitality, general health perceptions Cronbach’s alpha: 0.84
Cognitive complaints	MOS-CF6 [49, 73]	6 six-point Likert scale	Past week	0–100; higher scores indicated better cognitive functioning	Cronbach’s alpha: ≥ 0.89
	MDASI [50]	8 eleven-point Likert scale	Past 24 h	0–80; higher score indicates worse or more disturbing cognitive complaints	
Cancer worries	CWS [54]	8 + 1 four-point Likert scale	Past week	9–36; higher score indicates more frequent worries about cancer	Cronbach’s alpha: 0.87
Fatigue catastrophizing	FCS [55, 74]	10 five-point Likert scale	Current attitude	10–40; higher score indicates more catastrophizing	Cronbach’s alpha: 0.85
Self-efficacy	SES-28 [56, 75]	7 four-point Likert scale	Current attitude	7–28; higher score indicates higher level of perceived control over fatigue symptoms	Cronbach’s alpha: 0.68–0.77

*CES-D* Center for Epidemiological Studies – Depression scale, *CWS* Cancer Worry Scale, *FCS* Fatigue catastrophizing Scale, *MDASI* MD Anderson Symptom Inventory, *MFI* Multidimensional Fatigue Inventory**,** *MOS-CF6* Medical Outcomes Studies Cognitive functioning, *PSQI* Pittsburgh Sleep Quality Index, *SF-36* Medical Outcome Studies short form, *SES-28* Self-efficacy Scale 28, *STAI-6* State Trait Anxiety Inventory-6 items, *VAS* Visual Analogue Scale, *WSAS* Work and Social Adjustment Scale

A brief self-developed questionnaire will be used to examine the use of alcohol and caffeine, screen time prior to sleeping, solarium, wake-up lights, or the use of other interventions that could impact CRF (including physical exercise, CBT, or other interventions). Additional questions assess participant’s experience, compliance, and satisfaction with light therapy. Compliance is also assessed with a light therapy log during light therapy.

#### Actigraphy

Objective measures of sleep and circadian activity will be monitored with an accelerometer in a microelectromechanical system (MotionWatch8, Camntech, Cambridgeshire, United Kingdom). The MotionWatch8 is a small device, similar in size to a watch, with a tri-axial accelerometer. It has a 4.0 Mbits storage capacity and a waterproof casing. This watch will be worn on the non-dominant wrist for 10 (24-h) days at all measurement points and during light therapy. Output of the MotionWatch8 includes the following sleep parameters: time in bed, time out of bed, sleep onset latency (min), sleep efficiency, total time in bed (min), total sleep time (min), wake after sleep onset (min), number of awakenings, and average awakening time (min). Additionally, output of the MotionWatch8 includes the following circadian activity rhythm variables: interdaily stability (IS), Intra-Daily Variability (IV), Least 5 (L5) average, Most 10 (M10) average, and relative amplitude (RA). In addition, it offers nap analyses for naps during the day and day activity analyses.

An actigraphy log will be used to ensure that the scoring software of the actigraph detects the sleep habits of participants accurately. Based on the guidelines for the use of actigraphy, the following items will be included: bed time, attempted time to fall asleep, wake-up time, out-of-bed time, time of day time naps, times the actigraph was removed, unusual circumstances that might have affected sleep/wake patterns (such as illness) [45].

#### Biological samples

##### Salivary cortisol

All participants will be asked to collect saliva to assess cortisol on five different time points during 24 consecutive hours: 1) at personal waking time, 2) 30 min after awakening, 3) 45 min after awakening, 4) at 16.00 o’clock, and 5) at bedtime. These time points are chosen in line with published guidelines for determination of the *Cortisol Awakening Response* (CAR) [57]. The afternoon and evening samples are used to estimate the *diurnal cortisol slope* and the *area under the curve.*

Saliva will be collected by a passive drool technique into a propylene vial. Participants are not allowed to smoke, engage in vigorous exercise, eat or drink caffeinated drinks or food, and eat protein-rich meal during the sampling period starting 1 h prior to sampling. Eating and drinking of other nourishments is allowed until 5 min prior to sampling. Brushing of teeth is not allowed for 30 min before sampling. After sampling, the participant is instructed to record the time that they completed the sample and to refrigerate it. Samples will be returned to the study coordinator by mail after which the samples will be frozen at − 80 °C to keep samples stable until analysis. Cortisol levels will be determined with an electrochemiluminescensce immunoassay ‘ECLIA’ on the Cobas®6000 analyzer (Roche Diagnostics GmbH, Mannheim, Germany).

##### Salivary melatonin

A subgroup (*n* = 25 per condition) will be asked to collect five additional saliva samples in the evening to determine the *Dim Light Melatonin Onset* (DLMO). Starting point for this saliva collection will be 5 h prior to usual bedtime followed by one sample every sequential hour. Previous research indicated that these time points provide a reliable measurement for DLMO with at home collected saliva samples [58]. Participants receive the additional instruction to collect these samples in dim light conditions.

A commercial direct saliva melatonin radioimmunoassay (RIA; Bühlmann laboratories, Schönenbuch, Switzerland) will be used to assess melatonin levels in saliva. The DLMO will be determined based on a threshold of 4.0 pg/mL. Previous research indicated that a fixed threshold is the most convenient way to determine DLMO although there is a risk that DLMO cannot be determined in patients with sleep problems as a consequence of low secretion of melatonin [58]. When we address this problem in the current study, an alternative procedure will be used. DLMO will then be defined as the time when melatonin concentration is two SD above the basal mean of three daytime samples [59].

##### Blood samples

Blood samples are collected to measure biomarkers of inflammation and vitamin D at baseline. During T0 and T1, two tubes of 10 mL of blood will be collected. One of these tubes will be saved in the biobank NKI-AVL. The other will be used to assess vitamin D and the following inflammatory biomarkers in duplicate by ELISA: IL-1RA, hsIL-6, sTNF-RII, and hsCRP. Vitamin D has been associated with current levels of fatigue [60–62]. The before-mentioned biomarkers have previously been associated with fatigue in patients with cancer [35, 63]. The level of these biomarkers, as well as the change in biomarker levels will be used as parameters for the statistical analysis.

### Data management

The original signed informed consent forms are stored at the department of the participating institute where the participant is recruited. All participants receive a unique participant number, in order to code their outcome measures without the risk of harming anonymity. Participants can choose to complete an online or pen-and-paper version of the questionnaire. Paper versions of completed questionnaires and a (copy of) the signed informed consent forms are stored at the Division of Psychosocial Research and Epidemiology of the Netherlands Cancer Institute separately. Online completion of questionnaires will take place via an online secured (HTTPS) research tool, called Explora Zorg, which is specifically developed for research in Dutch health care. Each participant has a personal log-in code. Completed paper versions of the questionnaires will be entered in this online system by the research assistant.

The information given online by patients is accessible to the study staff only, via a secured code. This code is known by the principal investigator (EB), the study coordinator (DS), and the research assistant (JG). The principal investigator will safeguard the key to the code. The collected data in this research tool is saved on the secured database of the Netherlands Cancer Institute on a monthly basis.

Blood and saliva samples of all participants are stored at the general clinical laboratory of the Netherlands Cancer Institute. Each sample is coded with a unique participant number. Date and time of sampling are reported on the samples.

### Statistical methods

#### Sample size calculation

The MFI is the primary outcome on which sample size calculations are based. With a sample of 128 patients (*n* = 64 per group), the study will have an 80% power to detect an Cohen’s effect size of 0.5 for the main effect of light therapy on fatigue with a *p*-value set at 0.05 (power calculation with G*power 3 [64]). Cohen’s effect size of 0.5 means a 0.5 standard deviation difference on the primary measurement outcome, which is considered to be a clinical meaningful difference [65]. Participants who discontinue light therapy but complete questionnaires will be included in the intention-to-treat analysis.

#### Statistical analyses

Data will be analysed using the Statistical Package for the Social Sciences (SPSS). Although we endeavour to check all questionnaires upon their return and call participants to complete missing items, some data might still be missing. Missing values will be imputed according to the manual of the questionnaire. In general, descriptive statistics will be computed for the outcome variables, potential covariates and demographic variables. Bivariate analyses will be undertaken to explore associations between outcome and potentially confounding variables (e.g. season, diagnosis, years since diagnosis) using correlations (for continuous variables) and Chi-square tests (for categorical variables).

Group differences in change in fatigue during the trial will be investigated using a mixed effect growth model with random intercept and slope, nested within site (clusters of different hospitals). This approach takes into account the within and between person variability, and deals adequately with missing data [66]. If baseline differences are identified despite randomisation, these variables will be accounted for in the model. In case of non-ignorable dropout we will correct the model for different patterns of missing values [67]. All analyses will be done on an ´intention to treat´ basis. Additional explorative analyses will be done on a ´per protocol´ basis.

The mixed effect model approach described for change in fatigue will also be used to determine treatment effects of continuous secondary outcome measures. To evaluate between-group differences in categorical secondary outcome measures, we will use generalized estimating equations (GEE) for longitudinal data. This approach accounts for correlated within subject responses, allows for not normally distributed variables and deals adequately with missing data [67–69]. Since there are multiple outcomes, the *p*-values for each model will be adjusted for multiple comparisons.

Within the intervention group we will explore which variables are predictive for the efficacy of light therapy in reducing fatigue. A mixed effect model for longitudinal data will be used with fatigue as dependent variable and the following independent variables: sleep quality, depression, anxiety, cognitive complaints, quality of life, and biological circadian rhythms. The *p*-values will be adjusted for multiple testing.

### Monitoring

The Institutional Review Board of The Netherlands Cancer Institute did not appoint a data monitoring committee because of the low risk on adverse events. Instead, the investigator submits a summary of the progress of the trial to the accredited METC once a year. Information is provided on the date of inclusion of the first subject, numbers of subjects included and numbers of subjects that have completed the trial, serious adverse events/ serious adverse reactions, other problems, and amendments. Some study sites require adherence to local monitoring protocols.

## Discussion

CRF affects approximately 40 to 60% of long-term survivors treated for (non-) Hodgkin lymphoma. Recently, interest shifted to light therapy as a promising treatment for CRF. Previous studies showed a prevention of increasing levels of CRF in breast cancer patients during chemotherapy and a reduction of fatigue complaints in cancer survivors after exposure to BWL compared to exposure to dim red light. Yet, the patient samples in these studies were small and knowledge of possible mechanisms and long-term effect of light therapy is lacking. This trial investigates the efficacy of light therapy in survivors of HL and DLBCL and explores potential mechanisms explaining its efficacy, including chronobiological and psychosocial pathways.

This trial has several noteworthy strengths, including (1) the randomized controlled trial design; (2) recruitment in multiple centers across the Netherlands; (3) the use of a dim white light comparison condition instead of a dim red light comparison condition to exclude the influence of light color; (4) the use of intention-to-treat analyses; and (5) inclusion of long-term follow-up measurements to investigate the long-term efficacy of light therapy.

There are also several limitations in this trial. First, for practical reasons the duration of light therapy is 3,5 weeks in the current study while previous studies provided light therapy for 4 weeks. Since light therapy for CRF is an upcoming research field, the duration of light therapy and its efficacy is not yet investigated. Clinical practice suggests that the effect of light therapy is often seen within 2 weeks. If no effect is seen in this period, than it is unlikely to see a change in the following weeks. For this reason, it is expected that shortening the time period of light therapy with 4 days will not impact the efficacy of light therapy. Second, a somatic cause for fatigue complaints is an exclusion criterion. Yet, screening does not include assessments of possible somatic factors. Instead, the treating physician judges whether a patient has a somatic cause for fatigue or not. In case of doubt, a team of three experts will be consulted to judge whether someone can be included in the trial. Third, the DLMO is assessed with 5 saliva collections starting 5 h prior to sleep onset. Recommendations by EUCLOCK (a large European wide research network aiming to investigate the circadian clock in single cells and humans) advices to include a saliva collection until 1 h after sleep onset. Yet, this would influence someone’s sleep pattern and might affect fatigue levels the following day. For this reason, saliva is only collected prior to sleep onset.

In conclusion, new insights suggest the efficacy of light therapy as a treatment for cancer related fatigue. If proven to be effective, light therapy will provide an easy to deliver, low-cost and low-burden intervention, introducing a new era in the treatment of CRF. National implementation of light therapy will be facilitated via close collaboration with the BETER-clinics. Moreover, the investigation of potential mechanisms enriches the CRF literature with possible new suggestions for causative factors of CRF, a symptom that is neither well understood nor treated.

## Abbreviations

BWL: Bright white light
CBT: Cognitive-behavior therapy
CBTH: Cognitive behavior therapy with hypnosis
CES-D: Center for Epidemiological Studies – Depression scale
CRF: Cancer related fatigue
CWS: Cancer Worry Scale
DLBCL: Diffuse large B-cell lymphoma
DRL: Dim red light
DWL: Dim white light
FCS: Fatigue catastrophizing Scale
HL: Hodgkin lymphoma
MDASI: MD Anderson Symptom Inventory
MFI: Multidimensional Fatigue Inventory
MOS-CF6: Medical Outcomes Studies Cognitive functioning
PA: Physical activity
PSQI: Pittsburgh Sleep Quality Index
SCN: Suprachiasmatic nucleus
SES-28: Self-efficacy Scale 28
SF-36: Medical Outcome Studies short form
STAI-6: State Trait Anxiety Inventory-6 items
VAS: Visual Analogue Scale
WSAS: Work and Social Adjustment Scale
